# Current State of Infection Prevention in Missouri Long-Term Care Facilities

**DOI:** 10.1017/ash.2025.352

**Published:** 2025-09-24

**Authors:** Rachael Snyders, Brooklyn White, Christopher Blank, Cassandra Sherman, Megan Dethloff, Christine Zirges, Laken Chaney, Jonas Marschall

**Affiliations:** 1 Washington University School of Medicine; 2 Missouri Department of Health and Senior Services; 3 BJC HealthCare; 4 SSM Health; 5 University of Arizona College of Medicine – Phoenix

## Abstract

**Background:** A survey was conducted to understand the current state of infection prevention (IP) in Missouri (MO) long-term care facilities (LTCF) following the COVID-19 pandemic. The survey focused on staffing, training and education, and program elements. **Method:** The survey was developed and managed in Qualtrics™. It was distributed via email to 1468 individual email addresses for 1122 unique LTCF across Missouri in partnership with the Missouri Department of Health and Senior Services. The survey was available from July 29, 2024 through August 16, 2024. Survey results were anonymized; participants had the option to provide facility or contact information if desired for follow up. **Results:** The survey response rate was 11.1%, with 164 responses collected. Participants worked in LTCF categorized as long-term care, assisted living, memory care, short-term skilled nursing, short-term rehabilitation, residential, and behavioral health. The size of LTCF varied in numbers of residents and employees. Most respondents (82%) reported having a dedicated IP professional for their facilities, but only 2% stated they were allocated full-time to IP responsibilities. Job titles also varied widely. Over 50% of respondents stated their IP professionals had less than five years of IP experience, but 80% stated the person received specific training for the IP role.

Most respondents reported having policies for monitoring and improving hand hygiene, managing communicable illnesses, and screening for infectious diseases (ID). Nearly all respondents (99%) reported that policies remain for managing residents, employees, and visitors with COVID-19. However, most programs lack a dedicated budget for IP initiatives and a defined relationship with an ID clinician or healthcare epidemiologist. **Conclusions:** This is, to our knowledge, the first statewide survey of infection prevention in Missouri long-term care facilities. One limitation of this study is the small response rate, and another is a response bias in which facilities with robust IP programs are more likely to respond.

There are employees and programs dedicated to preventing infections in residents of MO LTCF. The workforce varies in IP experience, though considerable IP-specific training is occurring which fosters competency advancement. Lack of full-time allocation to IP responsibilities and variation in job titles may indicate role overlap of IP with other specialties. Limited funding and partnerships with ID clinicians may affect the scale and scope of IP programs. This survey demonstrated that IP resources are available in MO LTCF, and there may be opportunities to enhance their impact through increased staffing, funding, and professional collaborations.

